# The Placebo Effect in Medicine and Clinical Practice: A Narrative Review

**DOI:** 10.7759/cureus.91893

**Published:** 2025-09-09

**Authors:** Giorgos Tzigkounakis, Katerina Simati, Konstantinos Georgiadis

**Affiliations:** 1 Research, Health and Resilience Institute, Athens, GRC; 2 Medicine, National and Kapodistrian University of Athens, Athens, GRC

**Keywords:** acupuncture, conditioning, contextual effects, expectancy, musculoskeletal pain, neurobiological mediators, physiotherapy, placebo effect

## Abstract

Placebo effects are complex psychobiological phenomena driven by patients' expectations, conditioning, and contextual cues.

In clinical practice, placebo effects are mediated by endogenous opioid, dopaminergic, and cholecystokinin (CCK) pathways and are accompanied by characteristic alterations in brain connectivity. Functional imaging and neurochemical investigations converge to demonstrate that these are genuine neurophysiological phenomena rather than mere responses.

These effects are documented across pharmacologic, surgical, and physiotherapy and rehabilitation settings, including sham pills, surgery, and acupuncture. However, attributing observed improvements solely to placebo is challenging, as they may reflect natural history, regression to the mean, concomitant care, or active effects from control conditions.

This narrative review highlights the measurable influence and clinical relevance of placebo effects across interventions. Research should continue to clarify their mechanisms and triggers to strengthen causal inference in randomized trials and guide their ethical use in practice.

Accordingly, within an evidence-based and ethically sound framework, clinicians may purposefully leverage placebo-enhancing mechanisms to improve therapeutic outcomes, particularly in conditions where contextual and psychosocial factors strongly influence recovery. In practice, this entails transparent, constructive expectation-setting, minimization of nocebo cues, and, in selected contexts, the ethical use of open-label placebos alongside active treatment.

## Introduction and background

The placebo effect represents a complex psychobiological phenomenon wherein patients experience measurable improvements in symptoms after receiving an inert or nonspecific intervention.

Although no single universal definition exists, recent consensus guidance adopts working definitions that distinguish placebo treatments, placebo effects, and placebo responses. Placebo treatments are interventions lacking specific pharmacological activity, placebo effects refer to the psychobiological changes they elicit, and placebo responses denote the overall improvement observed in placebo groups, which also includes nonspecific factors such as natural history and regression to the mean [1,2]. Closely related is the concept of contextual effects, defined as outcome changes that arise from the therapeutic context rather than the intervention’s specific ingredients, including expectations, clinician behavior, treatment rituals, and the care setting [3].

In clinical research, placebos are typically defined by their lack of active pharmacological ingredients, serving as crucial controls in trials aimed at distinguishing specific treatment effects from nonspecific responses [4].

While initially linked to drug trials, placebo use has expanded to surgical procedures [5], manual therapy [6], psychiatry [7], and complementary and alternative medicine (CAM) [8]. Today, within evidence-based medicine (EBM), placebo-controlled, double-blind randomized controlled trials (RCTs) are considered the gold standard for evaluating any therapeutic modality [6,9,10].

Placebos have traditionally been viewed with skepticism, often associated with deception or patient manipulation. However, advances in neuroscience challenge this view, showing that placebo effects can activate specific neurotransmitter systems, including endogenous opioids, dopamine, and cholecystokinin (CCK) pathways [11]. This demonstrates that placebo effects are not merely psychological but also neurobiologically mediated, influencing outcomes across various medical conditions.

Real-world clinical practice further supports their relevance. Multinational surveys report routine use of placebos by doctors, mostly impure placebos such as vitamins or over-the-counter analgesics, often for pain, insomnia, or anxiety, with low patient disclosure and broad ethical acceptability [12-15]. Simulated encounters confirm that doctors’ beliefs and patient feedback shape treatment decisions [16].

The mechanisms behind placebo and nocebo effects involve complex cognitive and behavioral processes, including expectation, motivation, prior experience, and classical conditioning [17].

These have been demonstrated across diverse experimental and clinical contexts, ranging from pain and motor performance [11,18] to hormonal regulation [19] and optometric measurements [20], in both healthy volunteers and patient populations [19,21,22]. However, recent studies indicate that placebo responses may be less robust in healthy individuals, particularly in domains such as emotional processing or cognitive enhancement [23,24]. This underscores the influence of contextual factors and individual variability, while reinforcing the broader relevance of mind-body interactions in clinical care.

This narrative review aims to integrate conceptual perspectives on the historical development and mechanisms of the placebo effect with its clinical applications, highlighting their practical implications for clinical practice.

## Review

Methodology

This narrative review was conducted in accordance with the Scale for the Assessment of Narrative Review Articles (SANRA) quality criteria. A targeted, non-systematic search of PubMed and Scopus (≈2000-2025) was performed using combinations of the terms “placebo effect”, “nocebo”, “contextual effects”, “sham intervention”, “neurobiology”, and “clinical practice”. Earlier seminal works were also included for context. Reference chaining from key publications and triangulation across conceptual, mechanistic, and clinical sources were applied to broaden coverage. Inclusion was limited to peer-reviewed human studies and major reviews addressing mechanisms, neurobiology, or clinical applications. Gray literature and non-peer-reviewed reports were excluded. No formal risk-of-bias assessment, quantitative synthesis, or PRISMA flow diagram was undertaken, in keeping with the narrative review design.

Historical context of the placebo effect

The term placebo derives from the Latin *placere *(“to please”). Its earliest recorded use was in Saint Jerome’s fourth-century Vulgate translation of Psalm 116:9, where “I will walk before the Lord” became “Placebo Domino in regione vivorum,” meaning “I shall please the Lord in the land of the living” [25]. Scholars argue that this mistranslated the Hebrew, which emphasized walking before God [25,26]. The word later signified flattery and ritual comfort, especially in medieval funeral rites with “placebo singers” hired mourners [27]. In 1785, it entered medical literature in Motherby’s Medical Dictionary [28], and by 1811, Hooper’s Lexicon-Medicum defined it as “[any medicine] adapted more to please than to benefit the patient” [26]. From lexicographic definitions, scholarly attention then shifted to empirical investigation of mechanisms and clinical effects.

One of the earliest modern contributions to placebo research came from Beecher, who argued that placebo effects were not mere trial artifacts but active psychological mechanisms capable of triggering physiological responses [29]. Subsequent research has confirmed that the brain can influence bodily processes via expectancy, belief, and other cognitive-emotional mechanisms. Recognition of the mind’s role in healing, however, predates modern science. In classical Chinese Medicine, an early text advised: “If a patient does not consent to therapy with positive engagement, the physician should not proceed, as the therapy will not succeed” [30], a principle consistent with modern evidence linking placebo effects to patient-practitioner interactions, treatment context, and psychosocial cues. Placebo effects can extend beyond clinical care, with reviews showing moderate to large gains in endurance and even stronger nocebo impairments, highlighting the bidirectional power of expectation [31].

Mechanisms of the placebo effect

Two principal frameworks explain placebo effects, expectancy theory and classical conditioning [32].

Expectancy and conditioning are often framed as competing perspectives [33], yet they can operate jointly [34]. This co-action has been empirically demonstrated in experimental paradigms of pain and motor performance, with evidence indicating that combining verbal expectation with conditioning produces stronger placebo effects than either manipulation alone, a finding further substantiated by systematic reviews [35].

Recent experimental work has refined this view by showing that the strength of placebo hypoalgesia depends on order and congruence, with verbal suggestion preceding conditioning producing the most robust effects, while incongruent procedures tend to follow the direction of the last intervention [36]. Furthermore, adding observational learning, which refers to the acquisition of expectancies by observing others’ responses, to these two mechanisms may produce even stronger placebo effects in the context of analgesia, underscoring the value of combining multiple strategies [37].

Expectancy theory

Expectancy theory holds that placebo effects are mediated by explicit, consciously accessible expectancies [38]. A placebo produces change because the recipient anticipates that it will. This framework highlights the role of the psychosocial context in which treatment occurs [39], including the therapeutic relationship, sociocultural influences, and even the provider’s expectations [38].

Contemporary models situate expectation within Bayesian predictive processing, where strong prior beliefs bias perception and shape placebo or nocebo responses, sometimes even outside conscious awareness [40].

Classical conditioning

Classical, or Pavlovian, conditioning refers to the process by which a neutral cue, through repeated pairing, elicits a conditioned response. First described by Ivan Pavlov in 1927, his experiment with dogs demonstrated that salivation could be triggered by a bell previously associated with food [41]. 

For nearly a century, conditioning research has informed the etiology and treatment of anxiety disorders and chronic pain [42,43]. In chronic pain, altered responses to conditioned stimuli, such as diminished differential learning and heightened muscular reactivity, suggest a dysregulated associative learning process contributing to pain-related disability [43]. Conditioning principles have also been extended beyond psychology as recent work in molecular biology and DNA computing shows even biochemical systems can be engineered to display conditioning-like learning, underscoring its fundamental role across biological levels [44].

Applied to placebo effects, the placebo intervention is the conditioned stimulus, and the clinical improvement is the conditioned response. Numerous studies support this framework [45,46]. Voudouris et al. reported that conditioning can exceed verbal expectation in eliciting placebo effects [46]. Montgomery and Kirsch showed that conditioning enhances placebo effects, but that explicit expectation can override them [47]. Benedetti and colleagues confirmed this pattern in conscious functions such as pain and motor performance, where verbal suggestions could counter conditioning. In the same study, contrary suggestions did not change cortisol or growth hormone, while preconditioning with sumatriptan still produced the expected placebo-induced hormonal changes [17]. The authors proposed that expectancy mainly governs conscious functions, and that conditioning often predominates in unconscious functions such as hormonal release. However, later evidence complicates this separation. Yale’s “Mind over Milkshakes” study found that ghrelin responses were driven not by the actual nutritional content of a shake but by the belief that it was high or low calorie, showing that expectations may also shape hormonal activity [19].

Neurobiological work clarifies these routes. Amanzio and Benedetti showed that expectation cues and morphine conditioning induce naloxone reversible analgesia, which implicates endogenous opioids [34]. In parallel, ketorolac (NSAID) conditioning produced placebo effects that were partly naloxone-insensitive, which is consistent with non-opioid pathways. These results indicate that expectation reliably engages opioid mechanisms, while conditioning may recruit subsystems consistent with the pharmacodynamics of the conditioned drug. Thus, placebo analgesia can arise via distinct neurobiological pathways, which may be drug-specific and context-dependent.

From a cognitive perspective, some authors view conditioning trials as expectancy manipulations, so classical conditioning can operate as an experiential route to expectation formation, whereby repeated pairings link a cue to an outcome and the later inert cue elicits the expected effect [48].

Contextual and open-label modulators

While placebos are usually administered without disclosing their inert nature, Kaptchuk et al. tested an open-label placebo in irritable bowel syndrome (IBS) [49]. Participants were told they would receive inert “placebo pills” that prior studies linked to symptom relief through mind-body processes. A no-treatment control group allowed separation of placebo effects from the natural course of IBS. The placebo group reported nearly twice the symptom relief. Despite the small sample (n = 80) and lack of long-term follow-up, the findings were notable, leading the authors to call for further research on open-label placebos within ethical informed consent.

Subsequent systematic reviews and meta-analyses in chronic musculoskeletal pain [50] and chronic low back pain [51] have reported small-to-moderate improvements in self-reported pain and function, although the certainty of evidence was low and objective outcomes largely unaffected. By contrast, a trial in healthy volunteers found no benefits for cognitive performance, suggesting that open-label placebo effects are context-dependent and more likely to emerge in clinical populations than in healthy individuals [24].

Taken together, these studies imply that expectancy can play a role in open-label placebo responses, but its influence depends on clinical context and is not the sole driver. Other modulators include memory, motivation, and sensory cues [52]. For example, pill color can alter perceived and actual outcomes, with blue often linked to sedation, red and orange to stimulation, and white to pain relief [53].

Neurobiological mediators

Placebo and nocebo effects engage opioid, cannabinoid, dopaminergic, and CCK systems, shaped by expectation and learning.

Expectation-driven analgesia is often opioid-dependent, shown by naloxone reversal, beta-endorphin increases, and PET evidence of mu opioid engagement in cortical and limbic regions [54-56]. Conditioning with non-opioid drugs recruits endocannabinoids, and blockade with the CB1 antagonist rimonabant prevents NSAID-conditioned placebo analgesia [57].

The CCK system counteracts opioid effects and limits placebo responses. Proglumide, a CCK antagonist, enhances placebo analgesia and reduces nocebo hyperalgesia, while pentagastrin, a CCK-B agonist, induces anxiety and supports the involvement of CCK in nocebo mechanisms [58-60].

Dopamine plays a complex role. In the nucleus accumbens, release rises with both expected value and prediction error during placebo responses [40,56,61], and expectations about caffeine or conditioned cues can elicit striatal surges comparable to drug exposure [62,63]. Yet, neither sulpiride nor levodopa reliably alters placebo analgesia [64], indicating that dopamine more likely reflects the outcomes of expectation than directly drives them.

Neuroimaging converges with these pharmacologic data. fMRI and PET implicate the prefrontal cortex, anterior cingulate, insula, striatum, and periaqueductal gray, revealing connectivity changes within descending pain modulatory circuits [65]. Resting-state markers may further identify individuals prone to placebo responses [66]. fNIRS shows prefrontal and sensorimotor oxygenation changes aligned with suggested onset times [67], and EEG reveals placebo-related increases in frontal alpha asymmetry moderated by motivational traits [68].

Overall, mechanisms vary with induction method, prior learning, and individual network architecture, helping explain heterogeneous results across studies.

Clinical applications and evidence

Clinical applications of placebo effects are evident in the therapeutic relationship, surgery, physiotherapy, and complementary medicine, illustrating their broad relevance across different domains of healthcare.

Doctor-patient communication and expectations

Empathic, patient-centered communication with positive but realistic framing has been shown in healthcare consultations to reduce anxiety and improve symptoms [69]. Warmth, attentiveness, and supportive interaction further enhance outcomes, with the patient-practitioner relationship emerging as the most consistent contributor to the placebo response [70].

Communication about treatment itself is also important. Open-label placebo trials indicate that when the therapeutic rationale and ritual are explained clearly, patients may still experience meaningful benefits without deception [49-51]. Setting expectations should additionally address the likely time to benefit. Studies on sustained pain demonstrate that brief, specific cues about when improvement will begin can shift the onset of placebo analgesia, while analogous instructions can accelerate nocebo hyperalgesia [71,72].

Sham-controlled surgery

Placebo effects extend beyond drugs to almost any intervention, including surgery. Despite ethical concerns, many authors argue that well-designed sham-controlled trials are both feasible and valid for assessing surgical efficacy [73,74].

A landmark RCT by Moseley et al. found no greater benefit of arthroscopic debridement or lavage over sham surgery for knee osteoarthritis. At 24-month follow-up, all groups, including the sham arm with only skin incisions and simulated debridement, reported similar improvements in pain and function [75]. Subsequent evidence reinforces this pattern. A 2014 systematic review of 53 sham-controlled surgical trials showed improvement in 74% of placebo arms. In over half the studies (51%), outcomes were indistinguishable between sham and surgery, and surgery was clearly superior in less than half (49%), usually with small effect sizes [5]. Similarly, a 2017 systematic review of six orthopedic RCTs (n = 227) concluded that sham surgery was as effective as real procedures in reducing pain and disability [76].

Later trials confirmed these findings. A five-year follow-up of a multicenter RCT demonstrated no meaningful advantage of arthroscopic subacromial decompression over diagnostic (sham) arthroscopy or exercise therapy for shoulder impingement [77]. A 2024 double-blind RCT on sacroiliac joint fusion likewise found no significant benefit of surgery over a sham operation [78].

Health economic evaluations align with these findings. Data from the FIDELITY trial showed arthroscopic partial meniscectomy was not cost-effective compared with sham surgery, providing no additional quality-adjusted life years or cost savings [22].

The overall evidence underscores the strong placebo component in surgical interventions and the need for rigorous evaluation before their adoption or continued use.

Physiotherapy

Contextual factors in physiotherapy exert only small effects on pain and function, but they can account for almost 30% of the minimally clinically important difference [79]. This suggests they represent a clinically relevant element of treatment that can be ethically optimized.

Numerous studies highlight the impact of these contextual influences, as sham interventions often produced benefits beyond usual or minimal care. In chronic back and neck complaints, manual therapy and physiotherapy exceeded a sham of detuned ultrasound and diathermy, and the sham exceeded usual physician care [80]. Similar patterns appear in meniscal tear with osteoarthritis and in chronic low back pain, where both active and sham treatments produced comparable improvements, each superior to minimal care [81,82]. Large trials also report only small differences between active manipulation and sham [83], underscoring both the influence of contextual factors and the challenge of separating placebo from specific treatment effects in clinical physiotherapy.

Complementary and alternative medicine

CAM interventions are rich in ritual and contextual cues, and meta-research shows that about half of treatment effects in RCTs may stem from such factors rather than the specific therapy [84].

Homeopathy and Reiki illustrate how ritual alone may shape outcomes. Despite lacking established biological mechanisms [85], recent reviews report symptomatic improvements in rheumatic disease and oncology for homeopathy [85,86] and small but significant benefits in anxiety and quality of life for Reiki [87,88]. These findings underscore that contextual and ritual factors, rather than specific mechanisms, may drive observed effects.

Among CAM modalities, acupuncture is the most extensively studied. Systematic reviews show that acupuncture frequently outperforms no treatment but offers only small or inconsistent advantages over sham [89-94]. This pattern may reflect active contributions from somatic stimulation and context in both arms, which compresses between-group differences and complicates attribution of efficacy.

Distinguishing placebo effects from alternative explanations

Not all improvements observed after sham or placebo interventions reflect genuine psychobiological placebo effects. Outcomes can also be shaped by the natural course of illness, regression to the mean, co-interventions, and bias from patients or investigators. Spontaneous recovery or symptom fluctuation may be wrongly attributed to a placebo if no untreated control arm is included. Regression to the mean can likewise mimic treatment effects [95], but randomization and strict entry criteria reduce this risk. Co-intervention bias arises when participants adopt additional treatments, such as dietary changes or complementary therapies, which influence both experimental and control groups. Patients may also report improvement to please clinicians, and investigators may unconsciously influence assessments [96]. Blinding and the use of objective measures such as biomarkers help minimize this risk.

A further complication is that some sham interventions can trigger active physiological responses. For example, in acupuncture, sham procedures involve non-acupoint needling or non-penetrating needles. However, the former may trigger diffuse noxious inhibitory control (DNIC) responses, and the latter may engage mechanoreceptors potent in eliciting biochemical or neural responses [97]. DNIC is a supraspinal analgesic process seen in humans and animals [98-100], and can be elicited by cold water [101], noxious heat [102], electrostimulation [103], lasers [104], and skin puncture, including acupuncture and electro-acupuncture [105]. Similar concerns apply to other physical CAM modalities where sham procedures involve tactile or noxious stimulation, such as spinal manipulation or massage, raising the risk that control arms are not inert.

Distinguishing genuine placebo effects from nonspecific physiological responses therefore remains a key methodological challenge.

To synthesize the evidence reviewed, Table 1 summarizes the principal domains of placebo research, mechanisms, neurobiology, modulators, clinical applications, confounders, ethical themes, and clinical relevance, along with representative references.

**Table 1:** Summary of mechanisms, cofounders, modulators, and clinical relevance of the placebo effect CAM: complementary and alternative medicine, CCK: cholecystokinin, DNIC: diffuse noxious inhibitory control.

Domain	Key Findings	Representative evidence
Mechanisms	Expectancy, conditioning, and contextual cues are mainly mediated by opioid, dopamine, and CCK pathways	Huneke et al. [1]; Nikolakopoulou et al. [2]; Cook et al. [3]; Gupta and Verma [4]; Wartolowska et al. [5]; Bialosky et al. [6]
Neurobiology	Placebo responses engage μ-opioid receptors, increase dopamine release in the ventral striatum, and involve predictable brain connectivity patterns	Huneke et al. [1]; Gupta and Verma [4]; Huneke et al. [7]; Kaptchuk [8]; Akobeng [9]; Rains and Penzien [10]; Pollo et al. [11]; Tilburt [12]; Héron [13]; Sherman and Hickner [14]; Howick et al. [15]
Modulators	Contextual and psychosocial cues (therapeutic framing, rituals, patient-practitioner relationship, timing, sociocultural factors) and sensory features such as pill color can enhance placebo effects. Open-label placebos may also provide benefit without deception	Piedimonte et al. [16]; Benedetti et al. [17]; Benedetti et al. [18]; Crum et al. [19]; Vera et al. [20]; Zubieta et al. [21]
Clinical applications	Placebo effects are demonstrated in sham surgery, physiotherapy, acupuncture, and CAM	Kalske et al. [22]; Huneke et al. [23]; Hartmann et al. [24]; Lemoine [25]; Czerniak and Davidson [26]; De Craen [27]; Bierman [28]; Beecher [29]; Maoshing [30]; Chhabra and Szabo [31]; Meyer [32]; Stewart-Williams and Podd [33]; Amanzio and Benedetti [34]; Blythe et al. [35]; Bajcar [36]; van Lennepet al. [37]; Vase et al. [38]; Colloca and Benedetti [39]; Camerone et al. [40]; Jarius and Wildemann [41]; De Houwer [42]
Confounders	Improvements after placebo may reflect natural history, regression to the mean, co-interventions, or reporting bias. Additionally, not all sham interventions are inert, since true analgesia can arise from DNIC mechanisms	Rains and Penzien [10]; Harvie et al. [43]; Nakakuki et al. [44]; Voudouris et al. [45]; Voudouris et al. [46]; Montgomery and Kirsch [47]
Ethical considerations	Open-label placebos provide benefit without deception, and surveys reveal that physicians commonly use placebos	Piedimonte et al [16]; Benedetti et al. [17]; Ayers et al. [48]; Kaptchuk et al. [49]; Borg et al. [50]; Flávio-Reis [51]
Clinical relevance	Placebo mechanisms may be ethically integrated with evidence-based treatments to improve adherence, engagement, and outcomes in practice	Ayers et al. [48]; Price et al. [52]; Buckalew et al. [53]; Lipman et al. [54]

Strengths and limitations

This review is based on a targeted, non-systematic search, so it may not capture all relevant studies and does not include a formal risk-of-bias assessment or quantitative synthesis. To mitigate this, reference chaining from key publications and triangulation across conceptual, mechanistic, and clinical sources were used to identify additional relevant material. Conclusions therefore reflect conceptual integration and convergent trends from multiple high-quality sources rather than exhaustive coverage.

At the same time, the review brings together historical, mechanistic, and neurobiological perspectives with clinical applications. The emphasis on contextual factors, open-label placebo use, and practical implications offers an applied and ethically grounded perspective that adds value beyond purely experimental summaries.

## Conclusions

The placebo effect is a potent therapeutic phenomenon, though its scope remains incompletely understood. It can be elicited through expectation, conditioning, and learning, and accurate interpretation requires ruling out confounding factors before attributing changes to a true psychobiological response.

The patient’s mind, emotions, and the therapeutic relationship are central, with words, rituals, and context capable of altering brain chemistry and circuitry. Notably, placebo effects engage many of the same mechanisms as active drugs, implying cognitive-affective interactions with pharmacological action. Recognizing this, elements that amplify placebo effects, such as positive framing, symbolic acts, and culturally familiar practices, may be consciously and ethically incorporated into care. Used alongside evidence-based interventions, these strategies can improve engagement, adherence, and outcomes. Ultimately, placebo mechanisms should be ethically harnessed to enhance clinical benefit, not as substitutes for active treatment. Future systematic reviews and meta-analyses are important to assess the additional clinical impact of integrating placebo-related mechanisms into evidence-based medicine.
